# Correlation of the TIGIT-PVR immune checkpoint axis with clinicopathological features in triple-negative breast cancer

**DOI:** 10.3389/fimmu.2022.1058424

**Published:** 2022-12-05

**Authors:** Florence Boissière-Michot, Marie-Christine Chateau, Simon Thézenas, Séverine Guiu, Angélique Bobrie, William Jacot

**Affiliations:** ^1^ Translational Research Unit, Montpellier Cancer Institute Val d’Aurelle, Montpellier, France; ^2^ Biometrics Unit, Montpellier Cancer Institute Val d’Aurelle, Montpellier, France; ^3^ Department of Medical Oncology, Montpellier Cancer Institute Val d’Aurelle, Montpellier, France; ^4^ Institut de Recherche en Cancérologie de Montpellier (IRCM), Inserm U1194, Montpellier, France; ^5^ Faculty of Medicine, Montpellier University, Montpellier, France

**Keywords:** TIGIT, T-cell immunoglobulin and ITIM domain, PVR (CD155), triple negative breast cancer, immunohistochemistry, human tumor

## Abstract

**Background:**

T cell immunoreceptor with Ig and ITIM domains (TIGIT) interacts with poliovirus receptor (PVR) to contribute to cancer immune escape. Recently, TIGIT and PVR have been identified as promising immunotherapy targets. Their gene expression is upregulated in many solid tumors, but their protein expression level is not well documented, particularly in triple negative breast cancer (TNBC), the breast cancer subtype that most benefit from immunotherapy.

**Methods:**

TIGIT and PVR expression levels were assessed by immunohistochemistry in 243 surgically resected localized TNBC and then their relationship with clinical-pathological features and clinical outcome was analyzed.

**Results:**

TIGIT expression was observed in immune cells from the tumor microenvironment, whereas PVR was mainly expressed by tumor cells. High TIGIT expression was significantly associated with age (p=0.010), histological grade (p=0.014), non-lobular histology (p=0.024), adjuvant chemotherapy (p=0.006), and various immune cell populations (tumor infiltrating lymphocytes (TILs), CD3^+^, CD8^+^, PD-1^+^ cells; all p<0.0001), PD-L1^+^ tumor cells (p<0.0001), and PD-L1^+^ stromal cells (p=0.003). Infiltration by TIGIT^+^ cells tended to be higher in non-molecular apocrine tumors (p=0.088). PVR was significantly associated with histological grade (p<0.0001), the basal-like (p=0.003) and non-molecular apocrine phenotypes (p=0.039), high TILs infiltration (p=0.011), CD3^+^ (p=0.002), CD8^+^ (p=0.024) T cells, and PD-L1 expression in tumor (p=0.003) and stromal cells (p=0.001). In univariate analysis, only known prognostic factors (age, tumor size, lymph node status, adjuvant chemotherapy, TILs and CD3^+^ T-cell infiltrate) were significantly associated with relapse-free survival (RFS) and overall survival. High TIGIT and PVR expression levels tended to be associated with longer RFS (p=0.079 and 0.045, respectively). The analysis that included only non-molecular apocrine TNBC revealed longer RFS for tumors that strongly expressed TIGIT or PVR (p=0.025 for TIGIT and 0.032 for PVR).

**Conclusions:**

These results indicated that in TNBC, TIGIT^+^ cells can easily interact with PVR to exert their inhibitory effects. Their wide expression in TNBC and their association with other immune checkpoint components suggest the therapeutic interest of the TIGIT-PVR axis.

## Introduction

Triple-negative breast cancers (TNBC), which represent between 15 and 20% of all breast cancers, are characterized by the absence of hormone receptor (progesterone and estrogen) expression and HER2 overexpression. Therefore, they are not eligible for treatments against these targets. Until recently, surgery, chemotherapy and radiotherapy were the only possible therapeutic strategies for localized TNBC. The advent of immunotherapies could substantially change their management. Indeed, several intrinsic features suggest that TNBC might benefit from immunotherapy more than other breast cancer subtypes. First, TNBC are enriched in tumor infiltrating lymphocytes (TILs), and higher TILs density has been associated with better clinical outcome (1, 2) and response to neoadjuvant chemotherapy (2). Second, programmed cell death-ligand 1 (PD-L1), a target of immunotherapy agents, is overexpressed in TNBC compared with other breast cancers (3). Third, TNBC exhibit the strongest immunogenicity among breast cancer subtypes (4). All these elements form the rationale basis for the clinical development of immune checkpoint inhibitors in TNBC with contrasted results. For instance, the addition of the anti-PD1 (programmed cell death protein 1) antibody pembrolizumab to first-line chemotherapy and to neoadjuvant chemotherapy significantly improved the survival of patients with advanced PD-L1^+^ TNBC (5, 6) and of patients with early TNBC whatever their PD-L1 status (7–9), respectively. These results led to its approval by US and European agencies. On the other hand, results on the impact of adding the anti-PD-L1 monoclonal antibody atezolizumab to first-line chemotherapy are conflicting. The Impassion 130 study reported improved progression-free survival (PFS) when atezolizumab was associated with nab-paclitaxel in the overall cohort, whatever the PD-L1 expression. Moreover, a numerical improvement in overall survival (OS) was observed in the PD-L1^+^ TNBC population, without formal statistical testing due to the predefined statistical plan (10, 11). Conversely, the Impassion 131 study (12) on the association of atezolizumab with paclitaxel in the same setting did not find any evidence of PFS or OS improvement, whatever the PD-L1 expression level. Moreover, immunotherapy efficacy in TNBC is limited to a subset of patients, and some patients are resistant to such therapy. PD-L1 expression cannot be considered a robust biomarker, as demonstrated in the neoadjuvant setting where pembrolizumab benefit was independent of PD-L1 expression (7, 9). Thus, additional immunotherapy targets need to be identified in TNBC. Moreover, for immunotherapy success, robust biomarkers must be validated for rational patient selection and combination therapy personalization.

Recently, the T cell immunoreceptor with Ig and ITIM domains (TIGIT) receptor was identified as a promising immunotherapy target (13–15). TIGIT is an immune checkpoint protein expressed at low levels by T-cell subpopulations (memory CD4^+^ T cells, regulatory T cells, CD8^+^ T cells) and natural killer (NK) cells where its expression is upregulated after their activation. TIGIT interacts with several receptors on antigen-presenting cells (e.g. dendritic cells and macrophages) as well as on cancer cells and tumor microenvironment cells. Specifically, TIGIT binds with high affinity to poliovirus receptor (PVR, also named CD155) and with low affinity to nectin-2/CD112 and nectin-3/CD113 (16). PVR has a role in tumor cell invasion and migration (17). Upon binding to PVR, TIGIT induces an inhibitory signal directed to receptor- and ligand-expressing cells. It suppresses T cell activation and inhibits T and NK cell cytotoxicity by competing with CD226, another PVR ligand that activates T and NK cells. However, because TIGIT affinity for PVR is greater than that of CD226, TIGIT inhibitory role is dominant relative to CD226 activation signal (18). Thus, TIGIT is a marker of exhausted T cells in the tumor microenvironment (19), and its targeting by therapeutic antibodies could reverse T and NK cell exhaustion. In several preclinical models, blocking the PVR-TIGIT axis restored the anti-tumor immunity through T and NK cell activation and regulatory T cell inhibition (20). In addition, PVR-TIGIT axis inhibition has a synergistic action with PD1-PD-L1 axis blockade (21). Based on these results, the clinical evaluation of TIGIT blockade has been started in various cancer types (22), as monotherapy or in combination with anti-PD1/anti-PD-L1 drugs. In TNBC, a phase 1b trial is currently evaluating the safety, efficacy, and pharmacokinetics of tiragolumab, a human anti-TIGIT monoclonal antibody, in combination with atezolizumab and chemotherapy (NCT04584112).

Transcriptomic analyses showed that both *TIGIT* and *PVR* are overexpressed in TNBC compared with other breast cancer types (4, 15, 23, 24). In a series of breast cancers, including HER2^+^ tumors and TNBC, *PVR* gene expression was associated with poor clinical outcome (15). Counter-intuitively, *TIGIT* gene expression was associated with improved recurrence-free survival (RFS) and OS in basal-like breast cancers (25), a subpopulation that predominantly includes TNBC, and also in a cohort of patients with all breast cancer types (24). To the best of our knowledge, only few data on TIGIT and PVR protein expression are available for breast cancer, particularly the TNBC subtype.

Therefore, in this study, we evaluated, by immunohistochemistry (IHC), the expression of TIGIT and its receptor PVR in a well characterized population of 243 early TNBC to assess their prognostic value and their relationships with other biomarkers, particularly T cell populations and the PD1 and PD-L1 immune checkpoint proteins.

## Materials and methods

### Tissue samples

The present study was approved by the Montpellier Cancer Institute Review Board (approval N° ICM-CORT-2021-10) and followed the Declaration of Helsinki guidelines. All patients were informed that their samples and associated clinical-biological data could be used, after anonymization, in research projects and were given the opportunity to object.

All specimens used in this study were taken from a single Biological Resource Center (Montpellier Cancer Institute; declaration number BB−033−00059, authorization number AC-2008-700). Samples from patients with unifocal, unilateral, non-metastatic TNBC (i.e. <10% of cells expressing progesterone and estrogen receptors by IHC and HER2 0, 1+ by IHC or 2+ by IHC and *ERBB2* non-amplified by *in situ* hybridization), without history of another invasive cancer in the previous 5 years, were selected for the study. All samples were from chemotherapy-naive patients. In total, 349 samples were arrayed in six TMA (two invasive cores of 1 mm in diameter/sample).

### Immunohistochemistry

For IHC, rabbit monoclonal antibodies against TIGIT (clone E5Y1W) and PVR (D8A5G) (both from Cell Signaling Technology) and 3 µm-thin TMA sections were used. Briefly, TMA slides were immersed in high pH target retrieval solution (Dako Agilent Flex Kit K8000) using a PT-Link module (Dako Agilent) for simultaneous deparaffinization, rehydration and antigen retrieval. Following endogenous peroxidase inhibition with EnVision™ FLEX Peroxidase-Blocking Reagent (Dako Agilent Flex Kit K8000), slides were incubated with the primary antibodies at room temperature for 30 min. A mouse anti-rabbit antibody (Dako Agilent Ref K800921) was used for TIGIT signal amplification. Dako EnVision™ FLEX/horseradish peroxidase (HRP) reagent (Dako Agilent Flex Kit K8000) (i.e. a dextran backbone to which up to 100 HRP molecules and up to 20 secondary anti-mouse and anti-rabbit antibody molecules have been coupled) was used, followed by incubation with 3,3’-diaminobenzidine as chromogen to reveal TIGIT and PVR expression. Then, sections were counterstained with hematoxylin (Dako Agilent Ref K8008), dehydrated, and mounted with permanent mounting medium. All slides were stained as a single batch to reduce experimental variability.

The detailed procedures and scoring for other biomarkers used in this study and extracted from previously studies using the same samples are described in the corresponding publications (26–33).

### Image acquisition and scoring procedures

Stained TMA slides were scanned with a Nanozoomer^®^ scanner (Hamamatsu) at the x20 objective. Digitalized slides were analyzed independently by two investigators (FBM and MCC who is a board-certified pathologist) in a blinded manner. Missing cores (du to the use of these TMA in previous projects), cores containing <20 cancer cells, and cores with significant artefacts were excluded. Finally, TIGIT and PVR expression levels were analyzed in 243 TNBC samples.

**Figure 1:**
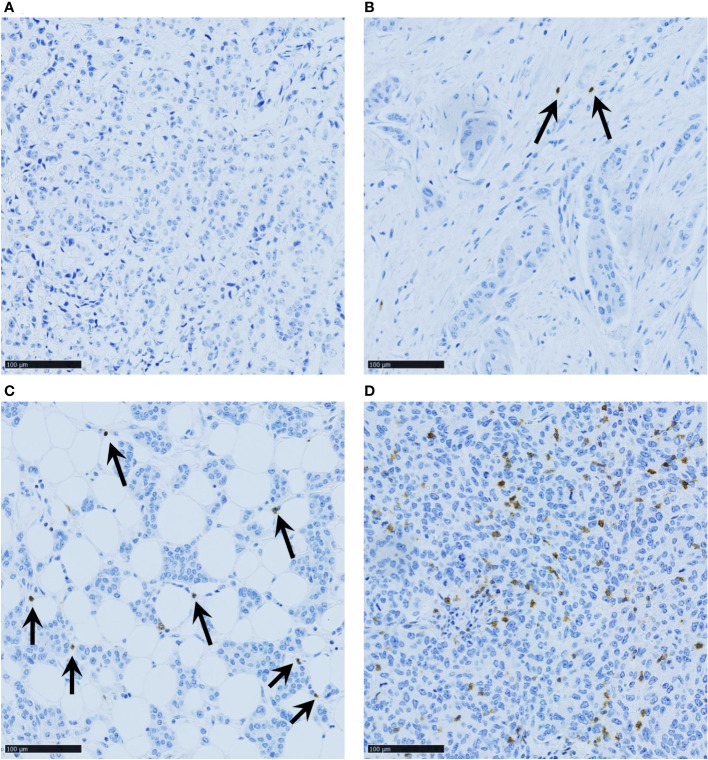
Representative images of TNBC samples with various TIGIT^+^ cell densities: **(A)** score 0 (no TIGIT^+^ cells in the whole core); **(B)** score 1 (few scattered TIGIT^+^ cells); **(C)** score 2 (core with some TIGIT^+^ cells, i.e. <20 TIGIT^+^ cells in a x40 field that represents 0.103 mm²); and **(D)** score 3 (at least one x40 field with ≥20 TIGIT^+^ cells). Scale bar 100µm.

TIGIT-stained samples were scored according to the TIGIT^+^ cell density: 0 = absence of TIGIT^+^ cells in the whole core; 1 = few scattered TIGIT^+^ cells; 2 = core with some TIGIT^+^ cells (i.e. <20 TIGIT^+^ cells in a x40 field that represents 0.103 mm²); and 3 = higher density (at least one x40 field with ≥20 TIGIT^+^ cells) (**Figure 1**). In case of scoring disagreement between observers (9% of cores), TIGIT status was determined after new joint analysis by the two investigators.

**Figure 2:**
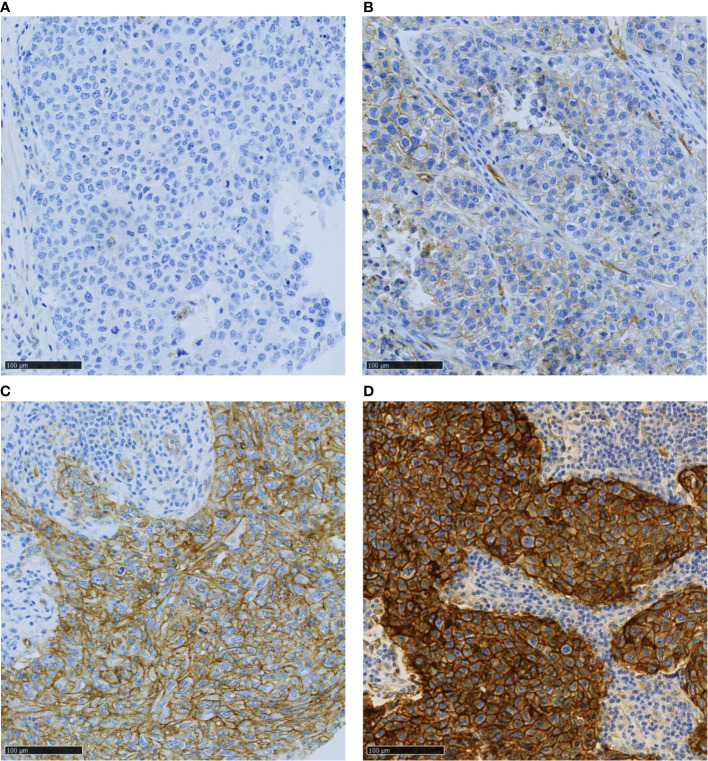
Representative images of TNBC samples with various PVR expression levels: **(A)** no, **(B)** weak, **(C)** moderate, and **(D)** strong signal intensity. Scale bar 100µm.

Tumor cell membrane PVR staining intensity was scored as negative (score 0), weak (score 1), moderate (score 2), or strong (3), and the percentage of positive cells was estimated at each intensity (**Figure 2**). A histochemical score (H-score), which ranged between 0 and 300, was obtained by adding each intensity score multiplied by its corresponding cell percentage. The H-score agreement between investigators was high (R²= 0.86, data not shown). The 10% most discordant cases were solved jointly. This increased the R² to 0.90. The mean H-score values were used for the analyses.

The basal-like phenotype was defined by the expression of EGFR and/or cytokeratin (CK) 5/6 in ≥10% of tumor cells, and molecular apocrine breast cancers by positive staining for androgen receptors and FOXA1 in ≥1% of tumor cells.

### Statistical analyses

Quantitative variables were described as number of observations (n), median, minimum, and maximum. For categorical variables, the number of observations (n) and percentages were computed excluding missing data. To investigate associations, univariate statistical analyses were performed using the Pearson’s chi-square or Fisher’s exact test, when applicable. Survival data were estimated with the Kaplan-Meier method, and presented as median and rate at 60 months, with 95% confidence intervals (CI). The median follow-up was assessed with the reverse Kaplan-Meier method and presented with its 95% CI. RFS was defined as the time from the date of surgery to the date of the first documented tumor relapse (local and/or distant). Patients alive without event were censored at the last known date to be alive. OS represented the time between the date of surgery and the date of death, whatever the cause. Survival curves were drawn and differences between groups were assessed with the log-rank test. Multivariate analyses were carried out using Cox proportional hazards regressions, with a stepwise selection procedure, to investigate known prognostic factors. Risk reduction was presented as hazard ratios (HR) with their 95% CI. The clinical factors associated with RFS in univariate analyses (p <0.2) were used to establish a combination of factors whose the prognostic impact was tested in multivariate analysis (Cox model). All p-values were two sided. The significance level was set at 5% (p <0.05). Statistical analyses were performed with the STATA 16.1 software (Stata Corporation, College Station, TX).

## Results

### Patients, tumor characteristics, and TIGIT/PVR expression

**Table 1** describes the main clinical-pathological features of the 243 TNBC samples in which TIGIT and/or PVR expression could be determined. Most samples were high-grade cancers (78.7%) of the ductal type (83.2%), mostly without lymph node involvement (65.1%). The patients’ median age was 58.2 years (range: 28.5 to 89.1) and 74.8% of them received adjuvant chemotherapy. Moreover, 67.6% of tumors were classified as basal-like, and 40% had a molecular apocrine phenotype.

**Table 1:** Clinicopathological features of the TNBC cohort.

	**N = 243**	**%**
**Age (years), median [min-max]**	58.2 [28.5-89.1]	
**Tumor size**		
T1	111	45.7
T2	116	47.7
T3/T4	16	6.6
**Lymph node status** (missing: 2)		
N-	157	65.1
N+	84	34.9
**Histological grade** (missing: 4)		
I/II	51	21.3
III	188	78.7
**Histology**		
Ductal	202	83.2
Lobular	12	4.9
Other	29	11.9
**Adjuvant chemotherapy** (missing: 1)		
No	61	52.2
Yes	181	74.8
**Basal-like phenotype** (missing: 2)		
Basal-like	163	67.6
Non basal-like	78	32.4
**Molecular apocrine phenotype** (missing: 13)		
Molecular apocrine	92	40.0
Non-molecular apocrine	138	60.0
**TILs** (missing: 6)		
<5%	105	44.3
≥5%	132	55.7
CD3* (missing: 3)		
Low	120	50.0
High	120	50.0
CD8* (missing: 5)		
Low	119	50.0
High	119	50.0
**PD-1** (missing: 13)		
0	57	24.8
]0-10[	60	26.1
[10-50[	92	40.0
≥50%	21	9.1
**PD-L1 (tumor cells)** (missing: 17)		
<1%	96	42.5
≥1%	130	57.5
**PD-L1 (stromal cells)** (missing: 20)		
0	39	17.5
]0-10[	69	30.9
[10-50[	63	28.3
≥50%	52	23.3
TIGIT* (missing: 1)		
Low	130	53.7
High	112	46.3
**PVR^§^** (missing: 3)		
Low	160	66.7
High	80	33.3

Low and high categories were defined according to the median*, or for PVR by grouping the two first terciles versus the third^§^ (see Material and Methods).

Consistent with previous studies (34, 35), TIGIT was exclusively expressed by stromal cells (**Figure 1**). Among the 242 assessable TNBC samples, 46.3%, 29.8%, and 19.4% displayed high (score = 3), moderate (score = 2), and low (score = 1) TIGIT^+^ cell density, respectively. In only 4.5% of samples (n=11/242), TIGIT^+^ cells could not be detected (score = 0). For correlation analysis, samples were split in two categories, according to the median score value: low TIGIT^+^ cell density (score ≤2) and high TIGIT^+^ cell density (score >2).

In total, PVR expression could be evaluated in 240 TNBC samples. PVR was mainly expressed at the tumor cell membrane. The H-score of PVR membrane expression in tumor cells ranged from 0 to 300 (**Supplementary Figure S1**), with a small subset of negative tumors (5%, n=12/240). Overall, the PVR H-score was low (>0-100), medium (>100-200), and high (>200) in 31.3%, 52.9%, and 10.8% of samples, respectively. For correlation analyses, the cohort was divided in two groups according to the second tercile value: low (H-score 0-≤148) and high (H-score >148) PVR expression. We did not observed a significant correlation between the expression of TIGIT and PVR, whether the data were expressed in 2 (**Table 2**, p = 0.215) or in 4 categories (**Supplementary Table 1**, p = 0.527).

**Table 2:** Correlations between TNBC clinicopathological features and TIGIT and PVR expression.

	TIGIT* Low	TIGIT* High	p-value	PVR^§^ Low	PVR^§^ High	p-value
	n (%)	n (%)		n (%)	n (%)	
TIGIT*						0.215
Low	–	–		89 (56.0%)	38 (47.5%)	
High	–	–		70 (44.0%)	42 (52.5%)	
Missing				1	0	
**Age (years)**			0.010			0.465
<58.2	55 (42.3%)	66 (58.9%)		78 (48.8%)	43 (53.8%)	
≥58.2	75 (57.7%)	46 (41.1%)		82 (51.3%)	37 (46.2%)	
			0.809			0.550
≤ 40	14 (10,8%)	11 (9.8%)		18 (11.3%)	7 (8.8%)	
≥40	116 (89.2%)	101 (90.2%)		142 (88.7%)	73 (91.2%)	
**Tumor size**			0.624			0.404
T1	56 (43.1%)	54 (48.2%)		73 (45.6%)	36 (45.0%)	
T2	64 (49.2%)	52 (46.4%)		74 (46.3%)	41 (51.3%)	
T3/T4	10 (7.7%)	6 (5.4%)		13 (8.1%)	3 (3.7%)	
**Lymph node status**			0.497			0.479
N-	82 (63.1%)	74 (67.3%)		106 (66.7%)	49 (62.0%)	
N+	48 (36.9%)	36 (32.7%)		53 (33.3%)	30 (38.0%)	
Missing	0	2		1	1	
**Histological grade**			0.014			<0.0001
I/II	35 (27.6%)	16 (14.4%)		46 (29.1%)	4 (5.1%)	
III	92 (72.4%)	95 (85.6%)		112 (70.9%)	74 (94.9%)	
Missing	3	1		2	2	
**Histology**			0.024			0.245
Other	14 (10.8%)	15 (13.4%)		18 (11.3%)	11 (13.8%)	
Ductal	105 (80.8%)	96 (85.7%)		133 (83.1%)	68 (85.0%)	
Lobular	11 (8.4%)	1 (0.9%)		9 (5.6%)	1 (1.2%)	
**Adjuvant chemotherapy**			0.006			0.079
No	42 (32.6%)	19 (17.0%)		45 (28.1%)	14 (17.7%)	
Yes	87 (67.4%)	93 (83.0%)		115 (71.9%)	65 (82.3%)	
Missing	1	0		0	1	
**Basal-like phenotype**			0.936			0.003
Basal-like	88 (67.7%)	75 (68.2%)		98 (62.0%)	65 (81.3%)	
Non basal-like	42 (32.3%)	35 (31.8%)		60 (38.0%)	15 (18.7%)	
Missing	0	2		2	0	
**Molecular apocrine phenotype**			0.088			0.039
Molecular apocrine	54 (45.0%)	37 (33.9%)		66 (44.0%)	23 (29.9%)	
Non-molecular apocrine	66 (55.0%)	72 (66.1%)		84 (56.0%)	54 (70.1%)	
Missing	10	3		10	3	
**TILs**			<0.0001			0.011
<5%	79 (61.7%)	26 (24.1%)		78 (50.3%)	26 (32.9%)	
≥5%	49 (38.3%)	82 (75.9%)		77 (49.7%)	53 (67.1%)	
Missing	2	4		5	1	
CD3*			<0.0001			0.002
Low	93 (73.2%)	27 (24.1%)		90 (57.0%)	28 (35.4%)	
High	34 (26.8%)	85 (75.9%)		68 (43.0%)	51 (64.6%)	
Missing	3	0		2	1	
CD8*			<0.0001			0.024
Low	86 (68.8%)	33 (29.5%)		87 (55.4%)	31 (39.7%)	
High	39 (31.2%)	79 (70.5%)		70 (44.6%)	47 (60.3%)	
Missing	5	0		3	2	
**PD-1**			<0.0001			0.460
0	44 (37.0%)	13 (11.8%)		40 (27.0%)	16 (20.3%)	
]0,10[	31 (26.1%)	28 (25.5%)		11 (7.4%)	10 (12.7%)	
[10,50[	38 (31.9%)	54 (49.1%)		58 (39.2%)	33 (41.8%)	
≥50%	6 (5.0%)	15 (13.6%)		39 (26.4%)	20 (25.2%)	
Missing	11	2		12	1	
**PD-L1 (tumor cells)**			<0.0001			0.003
<1%	65 (55.6%)	31 (28.7%)		73 (49.3%)	22 (28.6%)	
≥1%	52 (44.4%)	77 (71.3%)		75 (50.7%)	55 (71.4%)	
Missing	13	4		12	3	
**PD-L1 (stromal cells)**			0.003			0.001
0	25 (21.7%)	14 (13.0%)		32 (21.8%)	7 (9.3%)	
]0,10[	44 (38.3%)	25 (23.4%)		26 (17.7%)	26 (34.7%)	
[10,50[	29 (25.2%)	34 (31.8%)		36 (24.5%)	27 (36.0%)	
≥50	17 (14.8%)	34 (31.8%)		53 (36.0%)	15 (20.0%)	
Missing	15	5		13	5	

Low and high categories were defined according to the median*, or for PVR by grouping the two first terciles versus the third^§^ (see Material and Methods).

### Clinicopathological correlations with TIGIT and PVR expression

Assessment of the correlations between TIGIT expression and clinicopathological factors (**Table 2**) revealed a significant association between high TIGIT^+^ cell density and age <58.2 years (p=0.010, but not when using the 40-year cut-off), higher histological grade (p=0.014), non-lobular histology (p=0.024), and adjuvant chemotherapy (p=0.006). High TIGIT^+^ cell density was also strongly associated with high TILs infiltrate (p<0.001), assessed using Salgado’s criteria (36) or with CD3 (p<0.001) and CD8 (p<0.001) markers, and with strong expression of PD-1 on stromal cells (p<0.001) and of PD-L1 on tumor and stromal cells (p<0.001 and p=0.003, respectively). Ten of the eleven TIGIT-negative samples displayed low TILs infiltrate. TIGIT^+^ cell density was not associated with basal-like phenotype, but tended to be associated with tumors displaying a non-molecular apocrine phenotype (*i.e.* tumors that did not express androgen receptor and FOXA1, p=0.088).

High PVR expression was more often identified in tumors with high histological grade (p<0.001), basal-like phenotype (p=0.003), and non-molecular apocrine phenotype (p=0.039). High PVR expression on tumor cells was also associated with TILs (assessed with Salgado’s criteria (p=0.011), CD3 (p=0.002) or CD8 immunostaining (p=0.024)), and PD-L1^+^ tumor and stromal cells (p=0.003 and p<0.001, respectively). Lasty, PVR expression was higher (although not significant) in tumors from patients who received adjuvant chemotherapy (p=0.079). PVR expression was not correlated with age (with either the 40- or 58-year old threshold), tumor size, lymph node status, or PD-1 expression.

It should be noticed that, using the 4 TIGIT scores (null, low, moderate and high) or the 4 PVR groups (H score=0, >0-100, >100-200, >200-300), no additional significant correlations were found with clinico-pathological features compared to the analyses using TIGIT and PVR as dichotomized variables (low versus high), excepted for PVR for which a lack of expression was more often observed in non-ductal carcinomas (p=0.022; **Supplementary Tables 1 and 2**).

### Survival analyses

The median follow-up was 9.6 years (95% CI [9.0; 10.4]). During this period, 77 deaths (31.7%) and 65 (26.7%) relapses were recorded. Therefore, the 5-year OS rate was 78.5% (95% CI [72.7; 83.2]), and the 5-year RFS was 75.9% (95% CI [69.9; 80.9]). Most relapses occurred during the first 3 years of follow-up, which is consistent with the reported clinical course of TNBC (37, 38).

Univariate analysis showed that known prognostic factors (age <58.2-year old, tumor size, lymph node involvement, adjuvant chemotherapy, TILs or CD3^+^ cell infiltration) were significantly associated with OS and RFS (**Table 3**). Of note, no association with survival (OS: HR 1.48 (95%CI 0.63-3.49), p=0.370; RFS: HR 0.72 (95%CI 0.36-1.43), p=0.344) was seen with age, using an alternative threshold of 40-year old. This lack of significance could be linked to the imbalance between the 2 age groups, as patients ≤40-year old represented only 10.3% of the study population. Neither TIGIT^+^ cell density nor PVR expression was associated with OS, whether their levels of expression were analyzed in 2 (**Table 3**) or 4 categories (data not shown). However, when dichotomized in 2 groups of expression, high PVR expression was significantly associated with better RFS (p=0.045, **Table 3**) and TIGIT^+^ cell density tended to be associated with better RFS (p=0.079, **Table 3**). The combination of these two biomarkers, which interact with each other, did not bring any additional prognostic value (data not shown).

**Table 3:** Univariate analysis of clinicopathological variables associated with relapse-free survival and overall survival.

	Overall Survival			Relapse-Free Survival		
	HR	p	95% CI	HR	p	95% CI
**Age (years)**						
<58.2	1			1		
≥58.2	2.37	<0.001	1.47 - 3.84	1.69	0.039	1.03 - 2.79
≤40	1			1		
>40	1.48	0.370	0.63 - 3.49	0.72	0.344	0.36 - 1.43
**Tumor size**						
T1	1			1		
T2	2.47	0.001	1.47 - 4.17	2.18	0.006	1.25 - 3.80
T3/T4	5.96	<0.001	2.69 - 13.20	6.44	<0.001	3.09 - 13.40
**Nodal status**						
N-	1			1		
N+	2.30	<0.001	1.46 - 3.62	3.67	<0.001	2.24 - 6.02
**Histological grade**						
I/II	1			1		
III	0.84	0.498	0.51 - 1.39	0.94	0.835	0.54 - 1.65
**Histology**						
Ductal	1			1		
Lobular	0.71	0.570	0.22 - 2.30	1.19	0.725	0.45 - 3.15
Other	0.42	0.056	0.17 - 1.02	0.69	0.365	0.30 - 1.55
**Adjuvant chemotherapy**						
No	1			1		
Yes	0.30	<0.001	0.19 - 0.47	0.46	0.002	0.28 - 0.75
**Basal-like phenotype**						
Basal-like	1			1		
Non basal-like	0.91	0.711	0.56 - 1.48	0.78	0.324	0.78 - 2,12
**Molecular apocrine phenotype**						
Molecular apocrine	1			1		
Non-molecular apocrine	0.69	0.111	0.44 - 1.09	0.66	0.100	0.41 - 1.08
**TILs**						
<5%	1			1		
≥5%	0.47	0.001	0.30 - 0.75	0.42	0.001	0.25 - 0.70
CD3*						
Low	1			1		
High	0.58	0.020	0.36 - 0.92	0.55	0.021	0.33 - 0.92
CD8*						
Low	1			1		
High	1.02	0.929	0.65 - 1.60	0.86	0.532	0.53 - 1.39
**PD-1**						
0	1			1		
]0,10[	0.82	0.531	0.44 - 1.52	1.00	0.990	0.51 - 1.93
[10,50[	0.81	0.487	0.45 - 1.46	0.88	0.682	0.46 - 1.66
≥50%	1.06	0.887	0.47 - 2.39	0.98	0.962	0.39 - 2.48
**PD-L1 (tumor cells)**						
<1%	1			1		
≥1%	0.73	0.188	0.46 - 1.17	0.58	0.037	0.35 - 0.97
**PD-L1 (stromal cells)**						
0	1			1		
[10,50[	0.84	0.648	0.39 - 1.80	0.41	0.053	0.17 - 1.01
]0,10[	1.34	0.427	0.65 - 2.78	1.33	0.434	0.65 - 2.69
≥50%	0.91	0.807	0.41 - 2.0	0.83	0.646	0.38 - 1.82
TIGIT*						
Low	1			1		
High	0.69	0.115	0.44 - 1.09	0.64	0.079	0.39 - 1.05
**PVR^§^**						
Low	1			1		
High	0.07	0.135	0.41 - 1.13	0.56	0.045	0.31 - 0.99

Low and high categories were defined according to the median*, or for PVR by grouping the two first terciles versus the third^§^ (see material and methods).

In multivariate analysis, lymph node involvement, tumor size, and ductal histology were associated with shorter OS. Conversely, high TILs density and adjuvant chemotherapy were significantly associated with better OS (**Table 4**). Lymph node involvement, tumor size, low TILs infiltration, no adjuvant chemotherapy, and low PD-L1 expression on stromal cells were independent prognostic factors of shorter RFS (**Table 4**).

**Table 4:** Multivariate analysis of clinicopathological variables associated with relapse-free survival and overall survival.

	Overall Survival (n = 221)			Relapse-Free Survival (n = 214)		
	HR	p	95% CI	HR	p	95% CI
**Lymph node status**						
N-	1			1		
N+	2.30	<0.001	1.47 - 3.59	3.75	<0.001	2.14 - 6.56
**Tumor size**						
T1	1			1		
T2/T3/T4	2.27	0.002	1.34 - 3.87	1.84	0.051	1.00 - 3.38
**Histology**						
Ductal	1					
Other/Lobular	0.32	0.003	0.15 - 0.68	–	–	–
**TILs**						
<5%	1			1		
≥5%	0.43	0.001	0.27 - 0.70	0.52	0.021	0.30 - 0.91
**Adjuvant chemotherapy**						
No	1			1		
Yes	0.28	<0.001	0.18 - 0.44	0.44	0.002	0.27 - 0.74
**PD-L1 (stromal cells)**						
<10%				1		
≥10%	–	–	–	0.65	0.101	0.38 - 1.09

**Figure 3:**
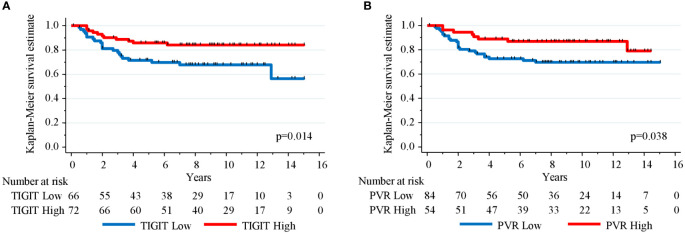
Kaplan-Meier curves showing relapse-free survival estimation in 138 TNBC samples with non-molecular apocrine phenotype (androgen receptor- and FOXA1-negative) stratified according to **(A)** TIGIT^+^ cell density and **(B)** PVR expression level.

Then, the prognostic impact of TIGIT and PVR expression in the non-molecular apocrine subgroup (n=138) was investigated because their expression was significantly (PVR) or almost significantly (TIGIT) associated with this subgroup. In this subgroup, the 5-year RFS rates were 71.6% (95% CI (58.8–81.1)) and 85.9% (95% CI (75.3-92.1)) (p=0.014) for patients with low and high TIGIT^+^ cell density expression, respectively (**Figure 3A**), and 72.6% (95% CI (61.4–81.1)) and 88.9% (95% CI (76.9–94.9)) (p=0.038) for patients with tumors showing low and high PVR expression, respectively (**Figure 3B**). Besides lymph node status, only high expression of TIGIT, PVR and PD-L1 on tumor cells remained independently associated with better RFS in multivariate analysis (**Table 5**).

**Table 5:** Multivariate analysis of clinicopathological variables associated with relapse-free survival in the non-molecular apocrine TNBC subgroup.

	Relapse-Free Survival (n = 134)		
	HR	p	95% CI
**Lymph node status**			
N-	1		
N+	2.62	0.009	1.27 - 5.39
TIGIT*			
Low	1		
High	0.41	0.025	0.19 - 0.89
**PVR** *^§^*			
Low	1		
High	0.39	0.032	0.16 - 0.92
**PD-L1 (tumor cells)**			
<1%	1		
≥1%	0.51	0.092	0.23 - 1.11

Low and high categories were defined according to the median*, and for PVR by grouping the two first terciles versus the third^§^ (see Material and Methods).

## Discussion

The aim of this study was to describe the expression of TIGIT and of its main receptor PVR, two potential immunotherapy targets, in a well-characterized cohort of 243 TNBC samples. Most TNBC samples displayed TIGIT expression, with expression restricted to stromal cells. TIGIT-immunoreactive cells could not be detected only in less than 5% of samples. Similarly, PVR was broadly expressed in malignant epithelium, and only 5% of the studied samples were PVR-negative. Our findings indicated that in TNBC, TIGIT (expressed in cells located in the microenvironment) can easily interact with its receptor PVR (expressed on tumor cells) to induce immunosuppression. Our IHC findings are in agreement with the high *TIGIT* and *PVR* gene expression observed in TNBC (4, 15, 23). To our knowledge, this study is the first to describe the clinicopathological correlations of TIGIT and PVR expression in the same series of breast cancers, specifically in TNBC. The study by Wang et al. reported PVR overexpression in TNBC compared with luminal breast cancers, but it included only 11 TNBC samples (23).

No published data supports a clear definition of high and low TIGIT or PVR expression in tumors, either in breast cancer or in other tumors. To increase the statistical strength, we dichotomized the population in two groups of high and low expression, based either on the median for TIGIT or on the two first terciles for PVR. Using these thresholds, we shown that, in accordance with some data presented in recent studies (23, 24), TIGIT and PVR expression levels were positively correlated with the presence of various cell populations in the tumor microenvironment, such as TILs and cells that express other immune checkpoint molecules (PD-L1, PD-1). This observation suggests the possibility of a multi-immune escape mechanism. Despite the strong inflammatory infiltrate, immune cells can become exhausted through expression of several immune checkpoints, and therefore cannot fight against cancer cells. This could explain why, as monotherapy, human monoclonal antibodies targeting PD-L1 (39) or PD-1 (40, 41) demonstrated only a modest clinical activity. This observation constitutes the rationale for the development of combined immunotherapies. In human colorectal cancer, it has been proposed that concomitantly targeting PD-L1 and TIGIT could restore intratumoral CD8^+^ T cell function (42). If confirmed, this combination could be also interesting for TNBC because in our cohort, high TIGIT expression was associated with high density of TILs, PD-1^+^ and PD-L1^+^ cells, and CD8^+^ T cells, suggesting that optimized combination strategies with several immune checkpoint inhibitors could reverse immune exhaustion. The efficacy of TIGIT blockade by therapeutic antibodies is currently investigated in several clinical trials, mainly using combination treatments (20, 43). Understanding the role of TIGIT and its partners in TNBC immunity could pave the way to tailored immunotherapy combinations. Therefore, it could be interesting to assess the expression of the activating receptor CD226 in TNBC because this PVR ligand plays a critical role in regulating the TIGIT-PD-1 blockade (16). In particular, PVR induces proteasomal degradation of CD226 in CD8+ TILs (44). CD226 downregulation could be a barrier to the effective targeting of this axis because it is required for enhancing the anti-tumor CD8^+^ T cell responses upon PD-1 and TIGIT blockade (45). Similarly, analysis of the expression of nectin-4, a ligand that is overexpressed in many tumor types (including breast cancer) and that exclusively interacts with TIGIT and not with CD226 (46), might give a more comprehensive picture of this axis.

The prognostic value of TIGIT and PVR is currently controversial. In our study, TIGIT and PVR protein expression had no prognostic value in the whole cohort of patients with TNBC. This in agreement with the study by Xie et al. (34) who did not find any correlation between TIGIT protein expression level and prognosis in 128 patients with breast cancer. However, the analysis did not focus on a specific breast cancer subtype. Other studies reported that *TIGIT* gene upregulation is associated with improved clinical outcome in a series of breast cancer (all types) (24), and in the basal subtype (25). Conversely, in a study on 197 breast cancer samples, high *PVR* mRNA levels were identified as an independent prognostic marker of shorter survival (15). However, this cohort combined all breast cancer subtypes, including HER2^+^ cancers and TNBC that represent the most aggressive breast cancer subtypes and showed the highest *PVR* mRNA levels (15), suggesting potential confounding variables. The present study found that non-molecular apocrine tumors more frequently expressed high PVR levels (and to a lesser extent also TIGIT). Moreover, in this TNBC subgroup, high expression of TIGIT or PVR was associated with better RFS. This suggests that the prognostic value of the TIGIT-PVR axis could be limited to specific breast cancer subtypes and reinforces the need for further investigation.

In summary, this study is the first to evaluate TIGIT and PVR protein expression in a large and well-characterized TNBC series. Their wide expression suggests that TIGIT interaction with PVR could hamper the anti-cancer immune surveillance. Moreover, the finding that their expression levels were associated with PD-1^+^ and PD-L1^+^ cells supports the development of combination strategies to concomitantly target the PD1-PD-L1 and TIGIT-PVR axes in this highly aggressive breast cancer subtype, particularly in non-molecular apocrine TNBC.

## Data availability statement

The raw data supporting the conclusions of this article will be made available by the authors, without undue reservation.

## Ethics statement

The present study was approved by the Montpellier Cancer Institute Review Board (approval N° ICM-CORT-2021-10) and followed the Declaration of Helsinki guidelines. All patients were informed that their samples and associated clinical-biological data could be used, after anonymization, in research projects and were given the opportunity to object.

## Author contributions

Study concept and design: FB-M and WJ. Experiments: FB-M. IHC analyses: FB-M, M-CC, SG, AB. Statistical analyses: ST. Drafting the manuscript: FB-M. Critical review of the manuscript: FB-M, SG, AB, WJ. All authors contributed to the article and approved the submitted version.

## Funding

This work was funded the the Montpellier Cancer Institute research fund.

## Acknowledgments

We acknowledge the Clinical Resources Center of the Montpellier Cancer Institute (CRB-ICM. no. BB-033-00059) and the imaging facility MRI, a member of the national infrastructure France-BioImaging infrastructure supported by the French National Research Agency (ANR-10-INBS-04. “Investments for the future”).

## Conflict of interest

The authors declare that the research was conducted in the absence of any commercial or financial relationships that could be construed as a potential conflict of interest.

## Publisher’s note

All claims expressed in this article are solely those of the authors and do not necessarily represent those of their affiliated organizations, or those of the publisher, the editors and the reviewers. Any product that may be evaluated in this article, or claim that may be made by its manufacturer, is not guaranteed or endorsed by the publisher.

## Supplementary material

Supplementary Figure 1 PVR expression (H-score) distribution in the 240 TNBC samples.
