# Supplementary material for: Correlation of the TIGIT-PVR immune checkpoint axis with clinicopathological features in triple-negative breast cancer
Source: Front Immunol. 2022 Dec 5;13:1058424. doi: 10.3389/fimmu.2022.1058424 (PMC9760730; doi:10.3389/fimmu.2022.1058424)
Supplement: Supplementary file 2 [file Table_1.docx]

**Supplementary Table 1:** Correlations between TNBC clinicopathological features and TIGIT expression

-----------------------------------------------------------------------------------

TIGIT expression

Null Low Moderate High

-----------------------------------------------------------------------------------

**PVR** (p=0.527)

Null 1 (9.1) 4 (8.9) 1 (1.4) 6 (5.4)

Low 4 (36.4) 17 (37.8) 19 (26.8) 35 (31.3)

Moderate 6 (54.5) 21 (46.7) 41 (57.7) 58 (51.8)

High 0 (0.0) 3 (6.7) 10 (14.1) 13 (11.6)

Missing 0 2 1 0

**Age**  (p=0.045)

<58.2 3 (27.3) 22 (46.8) 30 (41.7) 66 (58.9)

≥58.2 8 (72.7) 25 (53.2) 42 (58.3) 46 (41.1)

**Tumor size** (p=0.839)

T1 5 (45.5) 20 (42.6) 31 (43.1) 54 (48.2)

T2 6 (54.5) 22 (46.8) 36 (50.0) 52 (46.4)

T3/T4 0 (0.0) 5 (10.6) 5 (6.9) 6 (5.4)

**Lymph node status**  (p=0.544)

N- 8 (72.7) 32 (68.1) 42 (58.3) 74 (67.3)

N+ 3 (27.3) 15 (31.9) 30 (41.7) 36 (32.7)

Missing 0 0 0 2

**Histological grade** (p=0.004)

I / II 4 (36.4) 18 (39.1) 13 (18.6) 16 (14.4)

III 7 (63.6) 28 (60.9) 57 (81.4) 95 (85.6)

Missing 0 1 2 1

**Histology** (p=0.038)

Ductal 8 (72.7) 36 (76.6) 61 (84.7) 96 (85.7)

Lobular 0 (0.0) 5 (10.6) 6 (8.3) 1 (0.9)

Other 3 (27.3) 6 (12.8) 5 (6.9) 15 (13.4)

**Adjuvant chemotherapy** (p=0.003)

No 7 (63.6) 14 (30.4) 21 (29.2) 19 (17.0)

Yes 4 (36.4) 32 (69.6) 51 (70.8) 93 (83.0)

Missing 0 1 0 0

**Basal-like phenotype** (p=0.992)

Non basal-like 4 (36.4) 15 (31.9) 23 (31.9) 35 (31.8)

Basal-like 7 (63.6) 32 (68.1) 49 (68.1) 75 (68.2)

Missing 0 0 0 2

**Molecular apocrine phenotype** (p=0.255)

Molecular apocrine 5 (45.5) 22 (51.2) 27 (40.9) 37 (33.9)

Non molecular apocrine 6 (54.5) 21 (48.8) 39 (59.1) 72 (66.1)

Missing 0 4 6 3

**TILs** (p<0.001)

<5 10 (90.9) 32 (68.1) 37 (52.9) 26 (24.1)

≥5% 1 (9.1) 15 (31.9) 33 (47.1) 82 (75.9)

Missing 0 0 2 4

**CD3**  (p<0.001)

Low 10 (90.9) 41 (89.1) 42 (60.0) 27 (24.1)

High 1 (9.1) 5 (10.9) 28 (40.0) 85 (75.9)

Missing 0 1 2 0

**CD8**  (p<0.001)

Low 10 (90.9) 38 (82.6) 38 (55.9) 33 (29.5)

High 1 (9.1) 8 (17.4) 30 (44.1) 79 (70.5)

Missing 0 1 4 0

**PD1**  (p<0.001)

0 7 (70.0) 24 (55.8) 13 (19.7) 13 (11.8)

]0,10[ 3 (30.0) 11 (25.6) 17 (25.8) 28 (25.5)

[10,50[ 0 (0.0) 7 (16.3) 31 (47.0) 54 (49.1)

≥50% 0 (0.0) 1 (2.3) 5 (7.6) 15 (13.6)

Missing 1 4 6 2

**PD-L1 (tumor cells)**  (p<0.001)

<1% 10 (90.9) 25 (65.8) 30 (44.1) 31 (28.7)

≥1% 1 (9.1) 13 (34.2) 38 (55.9) 77 (71.3)

Missing 0 9 4 4

**PD-L1 (stromal cells)** (p=0.003)

0 5 (45.5) 5 (13.2) 15 (22.7) 14 (13.1)

]0,10[ 5 (45.5) 19 (50.0) 20 (30.3) 25 (23.4)

[10,50[ 1 (9.1) 9 (23.7) 19 (28.8) 34 (31.8)

≥50% 0 (0.0) 5 (13.2) 12 (18.2) 34 (31.8)

Missing 0 9 6 5

-----------------------------------------------------------------------------------

**Supplementary Table 2:** Correlations between TNBC clinicopathological features and PVR expression

-----------------------------------------------------------------------------------

PVR H-Score

0 >0-100 >100-200 >200-300

-----------------------------------------------------------------------------------

**TIGIT**  (p= 0.527)

Null 1 (8.3) 4 (5.3) 6 (4.8) 0 (0.0)

Low 4 (33.3) 17 (22.7) 21 (16.7) 3 (11.5)

Moderate 1 (8.3) 19 (25.3) 41 (32.5) 10 (38.5)

High 6 (50.0) 35 (46.7) 58 (46.0) 13 (50.0)

Missing 0 0 1 0

**Age**  (p= 0.302)

<58.2 3 (25.0) 41 (54.7) 64 (50.4) 13 (50.0)

≥58.2 9 (75.0) 34 (45.3) 63 (49.6) 13 (50.0)

**Tumor size**  (p= 0.939)

T1 5 (41.7) 36 (48.0) 58 (45.7) 10 (38.5)

T2 6 (50.0) 33 (44.0) 61 (48.0) 15 (57.7)

T3/T4 1 (8.3) 6 (8.0) 8 (6.3) 1 (3.8)

**Lymph node status** (p= 0.129)

N- 10 (83.3) 44 (59.5) 88 (69.3) 13 (52.0)

N+ 2 (16.7) 30 (40.5) 39 (30.7) 12 (48.0)

Missing 0 1 0 1

**Histological grade**  (p < 0.001)

I / II 10 (83.3) 20 (27.4) 19 (15.2) 1 (3.8)

III 2 (16.7) 53 (72.6) 106 (84.8) 25 (96.2)

Missing 0 2 2 0

**Histology** (p= 0.022)

Ductal 6 (50.0) 63 (84.0) 110 (86.6) 22 (84.6)

Lobular 2 (16.7) 5 (6.7) 3 (2.4) 0 (0.0)

Other 4 (33.3) 7 (9.3) 14 (11.0) 4 (15.4)

**Adjuvant chemotherapy** (p= 0.190)

No 6 (50.0) 19 (25.3) 29 (23.0) 5 (19.2)

Yes 6 (50.0) 56 (74.7) 97 (77.0) 21 (80.8)

Missing 0 0 1 0

**Basal-like phenotype** (p < 0.001)

Non Basal-like 10 (90.9) 26 (35.1) 35 (27.6) 4 (15.4)

Basal-like 1 (9.1) 48 (64.9) 92 (72.4) 22 (84.6)

Missing 1 1 0 0

**Molecular apocrine phenotype** (p= 0.001)

Molecular apocrine 9 (90.0) 33 (46.5) 40 (33.1) 7 (28.0)

Non molecular apocrine 1 (10.0) 38 (53.5) 81 (66.9) 18 (72.0)

Missing 2 4 6 1

**TILs** (p= 0.003)

<5 5 (45.5) 40 (54.1) 56 (45.5) 3 (11.5)

≥5% 6 (54.5) 34 (45.9) 67 (54.5) 23 (88.5)

Missing 1 1 4 0

**CD3** (p= 0.047)

Low 6 (54.5) 44 (58.7) 61 (48.8) 7 (26.9)

High 5 (45.5) 31 (41.3) 64 (51.2) 19 (73.1)

Missing 1 0 2 0

**CD8** (p= 0.015)

Low 9 (81.8) 45 (60.0) 54 (43.5) 10 (40.0)

High 2 (18.2) 30 (40.0) 70 (56.5) 15 (60.0)

Missing 1 0 3 1

**PD1** (p= 0.273)

0 5 (50.0) 23 (32.9) 24 (19.7) 4 (16.0)

]0,10[ 3 (30.0) 17 (24.3) 33 (27.0) 6 (24.0)

[10,50[ 2 (20.0) 25 (35.7) 53 (43.4) 11 (44.0)

≥50% 0 (0.0) 5 (7.1) 12 (9.8) 4 (16.0)

Missing 2 5 5 1

**%PD-L1 (tumor cells)**  (p < 0.001)

<1% 9 (100.0) 35 (50.0) 45 (37.2) 6 (24.0)

≥1% 0 (0.0) 35 (50.0) 76 (62.8) 19 (76.0)

Missing 3 5 6 1

**PD-L1 (stromal cells)** (p= 0.019)

0 2 (22.2) 15 (21.4) 19 (16.0) 3 (12.5)

]0,10[ 7 (77.8) 23 (32.9) 34 (28.6) 4 (16.7)

[10,50[ 0 (0.0) 21 (30.0) 35 (29.4) 7 (29.2)

≥50% 0 (0.0) 11 (15.7) 31 (26.1) 10 (41.7)

Missing 3 5 8 2

-----------------------------------------------------------------------------------
